# Pooled outcomes of performing freehand transperineal prostate biopsy with the PrecisionPoint Transperineal Access System

**DOI:** 10.1002/bco2.178

**Published:** 2022-06-28

**Authors:** Michael Tzeng, Spyridon P. Basourakos, Hiten D. Patel, Matthew J. Allaway, Jim C. Hu, Michael A. Gorin

**Affiliations:** ^1^ Department of Urology Weill Cornell Medicine New York New York USA; ^2^ Department of Urology Loyola University Medical Center Maywood Illinois USA; ^3^ Urology Associates and UPMC Western Maryland Cumberland Maryland USA; ^4^ Department of Urology University of Pittsburgh School of Medicine Pittsburgh Pennsylvania USA

**Keywords:** biopsy, diagnostic techniques, surgical, PrecisionPoint, prostatic neoplasms, sepsis, transperineal

## Abstract

**Objectives:**

To report the results of a pooled analysis evaluating the cancer detection rates, complications, and tolerability of prostate biopsies performed using the PrecisionPoint Transperineal Access System.

**Patients and Methods:**

The medical literature was reviewed to identify studies published prior to 1 October 2021 evaluating the PrecisionPoint device for performance of transperineal prostate biopsy. Pooled analyses were performed to assess overall and clinically significant cancer detection rates. Additionally, data on complications as well as patient tolerability of the procedure when performed under local anaesthesia were extracted.

**Results:**

Transperineal biopsy with the PrecisionPoint Transperineal Access System achieved overall and clinically significant cancer detection rates of 67.9% and 42.6%, respectively. Among patients with Prostate Imaging Reporting and Data System 3, 4, and 5 lesions on prostate magnetic resonance imaging, clinically significant disease was found in 31.7%, 55.7%, and 71.8% of patients, respectively. Complications were rare, with sepsis reported in 4 (0.1%) of 3411 procedures despite frequent omission of antibiotic prophylaxis. Patients reported acceptable tolerability of the procedure when performed under local anaesthesia.

**Conclusions:**

Within the available medical literature, there is uniform evidence supporting the use of the PrecisionPoint Transperineal Access System for performing prostate biopsy procedures. The reported cancer detection and infectious complication rates with this device are in line with other methods for performing transperineal prostate biopsy. A unique aspect of the PrecisionPoint device is its ability to facilitate performing transperineal prostate biopsy under local anaesthesia. This factor will likely lead to increased adoption of the beneficial transperineal approach to prostate biopsy.

## 1 INTRODUCTION

Compared with the transrectal approach, transperineal prostate biopsy offers fewer infectious complications^1^, ^2^ and superior sampling of the anterior prostate.^3^, ^4^, ^5^ Historically, the most widely used technique for performing transperineal prostate biopsy utilized a mechanical stepper unit equipped with a brachytherapy grid or similar needle guide.^6^ This technique requires the user to make multiple needle passes through the perineal skin and thus requires general or spinal anaesthesia for patient tolerability. As a result, this procedure has had relatively limited uptake by American urologists.^7^ To address this limitation, the freehand technique for performing transperineal prostate biopsy procedures was developed.^8^ With this technique, an access cannula is placed through the perineal skin, and the biopsy needle is passed repeatedly through this common entry site, thereby improving patient comfort and allowing for performance under local anaesthesia. However, a major shortcoming of this technique is the considerable technical skill required to correctly align the biopsy needle in the plane with the ultrasound probe while performing this procedure.^8^

In recent years, the limitations of the original freehand technique for performing transperineal prostate biopsy have been overcome by the introduction of a purpose‐built, probe‐mounted needle guide that includes a common access cannula known as the PrecisionPoint Transperineal Access System (Perineologic, Cumberland, MD).^5^, ^9^, ^10^, ^11^, ^12^, ^13^, ^14^, ^15^, ^16^, ^17^, ^18^, ^19^, ^20^, ^21^, ^22^, ^23^, ^24^ Following its approval by the United States Food and Drug Administration in 2016, this device facilitated the rapid adoption of transperineal prostate biopsy worldwide and led to widespread calls for the complete abandonment of the transrectal approach to prostate biopsy.^25^, ^26^ In this report, we provide the results of a systematic review of the available literature on the outcomes and complications of performing transperineal prostate biopsies using this novel device.

## 2 PATIENTS AND METHODS

This study was registered with PROSPERO, the international prospective registrar of systematic reviews (registration number CRD42022317894). A systematic search of the MEDLINE database was performed to identify studies investigating use of the PrecisionPoint device for performing transperineal biopsy of the prostate published before 1 October 2021. Studies reporting rates on prostate cancer detection and/or complications were included in our analysis. Titles and abstracts were screened for eligibility. Articles that were reviews, editorials, or written in non‐English were excluded. A meta‐analysis was conducted to determine the primary outcomes of overall (any grade) and clinically significant (grade group ≥2) cancer detection rates (CDRs). Additional subanalyses were performed to determine CDRs for biopsy‐naïve patients as well as those with known Prostate Imaging Reporting & Data System (PI‐RADS) score. Weights for meta‐analyses were based on random effects modelling with Freeman‐Tukey double arcsine transformation applied to stabilize variances for binomial data.^27^ Weights are primarily influenced by sample size but random effects meta‐analysis also incorporated between‐study variance into the denominator of each weight. Analyses were conducted using STATA v15.0 (STATA Corp, College Station, TX, 2017). Finally, data on complications and patient tolerability of the procedure under local anaesthesia were extracted and summarized.

For the primary outcomes of overall and clinically significant CDRs, publication bias was graphically assessed using funnel plots. Risk of bias was assessed based on the Agency for Healthcare Research and Quality (AHRQ) guidelines.^27^, ^28^ Two investigators independently rated the included studies considering three items: population (specifying details on patient biopsy history), study design (specifying details on biopsy template and MRI use), and consecutive enrolment. If all three items were rated favourable, the study was designated as high quality. If one item was unfavourable or unclear, the study was designated as moderate quality. If two or all three items were unfavourable or unclear, the study was designated as low quality. Strength of evidence was graded using the AHRQ Evidence‐based Practice Center Methods Guide for Conducting Effectiveness and Comparative Effectiveness Reviews scheme.^28^

## 3 RESULTS

**FIGURE 1:**
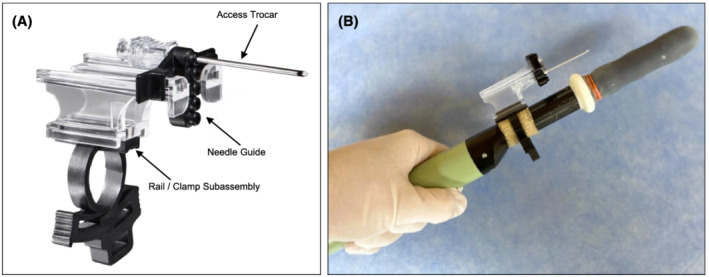
Images of the PrecisionPoint Transperineal Access System (Perineologic, Cumberland, MD). (A) The assembled PrecisionPoint device is composed of an access trocar, needle guide, and rail/clamp subassembly. (B) The PrecisionPoint device attached to a model 8658 biplanar transrectal ultrasound probe (BK Medical, Peabody, MA)

In total, 95 articles were identified using our search strategy. Of these, 17 (18%) met inclusion criteria and underwent full‐text analysis (Figure S1). One study^24^ was excluded from the pooled analysis of CDRs due to overlapping patients with a subsequently published report^13^ that included a larger sample size. However, only the earlier of the two reports included data on procedural complications and so was included in our analysis of this endpoint. Across the 16 studies included in the analysis of CDRs, data was available for 3886 transperineal biopsy procedures (Table 1). The included studies were performed in the United States (*n* = 8), the United Kingdom (*n* = 5), Asia (*n* = 2), and Australia (*n* = 1). All procedures were performed with similar overall technique with only minor variations. Detailed descriptions of the technique for performing biopsy procedures with the PrecisionPoint device can be found elsewhere.^19^, ^22^, ^29^, ^30^ Images of the device, including its use during MRI‐targeted prostate biopsy, can be found in Figure 1 and Figure S2. In total, 3420 (89%) men had a biopsy procedure under local anaesthesia.

**TABLE 1:** Patient characteristics and cancer detection rates of studies assessing the PrecisionPoint Transperineal Access System

Study	Sample size, *n*	Performed under local anaesthesia, *n* (%)	Age (years)	Total cores	Template	MRI Targeting	Patients with MRI targets, *n* (%)	Total MRI targets, *n*	Biopsy naive, *n* (%)	Prior negative, *n* (%)	Active surveillance, *n* (%)	Overall CDR, %	Clinically significant CDR, %
Chen et al. (2021)^9^	212	212 (100)	Mean 69.4 (SD 7.8)	Median 12 (R 4–38)	12‐core	Cognitive	39 (18.4)	NR	200 (94.3)	0	12 (5.7)	61.8	51.9
Hogan et al. (2021)^10^	40	40 (100)	Median 63.5 (IQR 10)	Median 33 (IQR 21.5)	NR	Cognitive	NR	NR	31 (77.5)	4 (10.0)	5 (12.5)	55.0	35.0
Islam et al. (2021)^11^	111	111 (100)	Median 63 (R 43–76)	NR	12‐core	No targets	0	0	90 (81.1)		21 (18.9)	53.2	38.7
John et al. (2021)^12^	313	313 (100)	Median 71 (IQR 66–75)	Antibiotics: Median 20 (IQR 18–22) No antibiotics: Median 23 (IQR 19–24)	Ginsburg	Cognitive or software (unspecified)	192 (61.3)	NR	NR	NR	NR	77.3	55.0
Kim et al. (2021)^13^	301	301 (100)	Median 67 (IQR 62–73)	Systematic: Median 20 (IQR 19–21) Targeted: Median 3 (IQR 3–4)	20‐core	Software (UroNav)	301 (100)	NR	193 (64.1)	21 (7.0)	87 (28.9)	79.1	49.2
Lopez et al. (2021)^14^	1218	1218 (100)	Median 68 (IQR 62–73)	Median 24 (R 1–47)	Ginsburg	Cognitive	684 (56.2)	NR	674 (55.3)	257 (21.1)	287 (23.6)	67.0	52.0
Meyer et al. (2021)^5^	279	279 (100)	Median 68 (IQR 64–72)	Median 12 (IQR 12–15)	12‐core	Cognitive	120 (43.0)	136	0	0	279 (100)	NR	21.2
Starmer et al. (2021)^15^	56	56 (100)	Mean 66.8 (R 53–80)	Median 22 (R 15–32)	NR	NR	NR	NR	13 (23.2)	25 (44.6)	18 (32.1)	NR	35.7
Szabo et al. (2021)^16^	242	242 (100)	Median 63 (R 29–93)	Median 20 (R 1–31)	12‐sector	Cognitive or software (KOELIS Trinity)	48 (19.8)	NR	111 (45.9)	106 (43.8)	25 (10.3)	43.4	14.0
Urkmez et al. (2021)^17^	289	5 (1.7)	Prostate cancer screening: Mean 64 (SD 7) AS: Mean 64 (SD 7.5)	Prostate cancer screening: Mean 28 (SD 1.5) AS: Mean 28 (SD 2.9)	28‐core	Cognitive	138 (47.8)	NR	0	125 (43.3)	164 (56.7)	73.7	47.8
Yuwono et al. (2021)^18^	72	72 (100)	Median 70 (R 46–83)	Median 24 (R 1–47)	Ginsburg	Cognitive	26 (36.1)	31	72 (100)		0	58.3	43.1
Gorin et al. (2020)^19^	95	88 (92.6)	Median 68.8 (R 52–86.4)	Systematic: Median 12 (R 12–14) Targeted: Median 1 (R 1–3)	12‐core	Cognitive	95 (100)	124	56 (58.9)		39 (41.1)	83.2	54.7
Kum et al. (2020)^20^	176	160 (90.9)	Mean 65 (R 36–83)	Systematic: Mean 24.2 (R 12–38) Systematic + Targeted: Mean 27.9 (R 16–41) Targeted: Mean 6.8 (R 6–13)	Ginsburg	Cognitive	85 (48.3)	NR	156 (88.6)	1 (0.6)	15 (8.5)	79.0	NR
Neale et al. (2020)^21^	282	163 (57.8)	Mean 66.8 (R 36–80)	Systematic: Mean 24 (R 5–42) Targeted: Mean 4.2 (R 1–11)	Modified Ginsburg	Cognitive	282 (100)	NR	230 (81.6)	0	52 (18.4)	84.0	69.0
Meyer et al. (2018)^22^	43	43 (100)	Median 62 (R 44–73)	Active surveillance: Median 14 Biopsy‐naive: Median 12	12‐core	No targets	0	0	31 (72.1)		12 (27.9)	48.8	16.3
Ristau et al. (2018)^23^	117	117 (100)	Median 70 (IQR 63–75)	Median 16 (IQR 14–20)	10‐sector	No targets	0	0	80 (68.4)	10 (8.5)	27 (23.1)	70.9	51.3

Abbreviations: AS, active surveillance; CDR, cancer detection rate; GA, general anaesthesia; IQR, interquartile range; LA, local anaesthesia; MRI, magnetic resonance imaging; NR, not reported; R, range.

**FIGURE 2:**
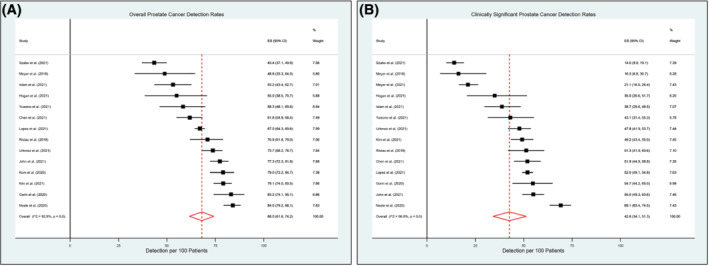
Forest plots for cancer detection rates of (A) overall and (B) clinically significant prostate cancer

In our pooled analysis, CDRs for overall and clinically significant disease were 68.0% (range: 43.4–84%) and 42.6% (range: 14–69.1%), respectively (Figure 2). Among the 1378 biopsy‐naïve patients across seven studies with available data, overall and clinically significant CDRs were 66.6% (range: 48.4–76.3%) and 54.5% (range: 35.5–61.5%), respectively (Figure S3). Magnetic resonance imaging (MRI) targeting with the PrecisionPoint device was performed in 12 (75%) studies, mainly using the visual estimation method, also known as cognitive fusion (Table 1). Among those reporting outcomes by PI‐RADS score, overall CDRs for patients with PI‐RADS 3, 4, and 5 lesions were 55.9%, 79.5%, and 89.1%, respectively (Figure S4A). Clinically significant disease among patients with PI‐RADS 3, 4, and 5 lesions were 31.8%, 55.7%, and 71.8%, respectively (Figure S4B).

Assessment of publication bias suggested bias towards lower overall CDRs in studies with fewer patients (Figure S5A). For the outcome of clinically significant CDR, there appeared to be low risk of publication bias (Figure S5B). Across the 16 unique studies that were evaluated for risk of bias, 5 (31%), 7 (44%), and 4 (25%) were identified as having a low, moderate, and high risk of bias, respectively. The strength of evidence was rated to be moderate due to medium study limitations, direct outcome measurement, consistent and precise event rates, and undetected reporting bias.

The analysis of complications included data from 3411 patients across 15 studies (Table 2). The complications of sepsis, urinary retention, and bleeding requiring medical intervention occurred in 0–2.5%, 0–4.7%, and 0–2.3% of patients, respectively. The raw pooled proportions of patients experiencing sepsis, urinary retention, and bleeding requiring medical intervention were 0.1% (4/3411), 1.3% (45/3411), and 0.2% (7/3411), respectively. Tolerability of the biopsy procedure was assessed using quantitative pain scales or qualitative surveys in seven studies with data from 765 patients. Four studies evaluated tolerability using the Visual Analog Scale (VAS). The median overall pain for the procedure ranged from 2.8 to 4.5.^9^, ^15^, ^16^, ^20^ One prospective study compared tolerability between transrectal prostate biopsy and the transperineal approach with PrecisionPoint and found no difference in VAS scores between the two groups.^15^ In three studies, surveys from patients with experience undergoing both a transrectal and transperineal prostate biopsy suggested that the transperineal approach may be equally or more preferable.^15^, ^16^, ^19^

**TABLE 2:** Reported complication rates for transperineal biopsy using the PrecisionPoint Transperineal Access System

Study	*n*	Prophylactic antibiotics routinely used?	Patients receiving antibiotics, *n*	Antibiotic type	Infections requiring IV antibiotics, *n* (%)	Sepsis, *n* (%)	Urinary retention, *n* (%)	Bleeding complicated by clot retention, transfusion, or corrective invasive procedure, *n* (%)
Briggs et al. (2021)^24^	130	Yes	62	NR	0	0	2 (1.5)	1 (0.8)
Chen et al. (2021)^9^	212	Yes	200	Cephalexin	0	0	8 (3.8)	0
Hogan et al. (2021)^10^	40	Yes	20	Cefazolin and Gentamicin or Ciprofloxacin	1 (2.5)	1 (2.5)	1 (2.5)	0
Islam et al. (2021)^11^	111	No	0	N/A	1 (0.9)	0	1 (0.9)	0
John et al. (2021)^12^	313	Yes	149	Ciprofloxacin or Gentamicin	1 (0.3)	1 (0.3)	0	1 (0.3)
Lopez et al. (2021)^14^	1218	Yes	1106	Ciprofloxacin, Co‐amox, Cephalexin, or Gentamicin	2 (0.2)	2 (0.2)	20 (1.6)	0
Starmer et al. (2021)^15^	56	Yes	56	Gentamicin	0	0	0	0
Szabo et al. (2021)^16^	242	Yes	30	Ceftriaxone or Ciprofloxacin	1 (0.4)	0	1 (0.4)	3 (1.2)
Urkmez et al. (2021)^17^	304	No	0	N/A	0	0	4 (1.3)	0
Yuwono et al. (2021)^18^	72	Yes	72	Cefuroxime	0	0	2 (2.8)	0
Gorin et al. (2020)^19^	95	No	1	N/A	0	0	1 (1.1)	0
Kum et al. (2020)^20^	176	Yes	176	Gentamicin	0	0	1 (0.6)	1 (0.6)
Neale et al. (2020)^21^	282	NR	NR	NR	0	0	0	0
Meyer et al. (2018)^22^	43	No	0	N/A	0	0	2 (4.7)	1 (2.3)
Ristau et al. (2018)^23^	117	No	0	N/A	0	0	0	0

*Note*: Meyer et al. (2021)^5^ did not report complications.

Abbreviation: NR, not reported.

## 4 DISCUSSION

In this report, we provide the results of a systematic review detailing the outcomes of performing transperineal prostate biopsy using the PrecisionPoint Transperineal Access System. The use of this device overcomes the limitations of previously described methods of performing transperineal prostate biopsy and has facilitated the adoption of this approach to prostate biopsy, which can now readily be performed under local anaesthesia.

In our analysis, the pooled CDRs for overall and clinically significant prostate cancer were 68.0% and 42.6%, respectively. These rates are consistent with previous reports on transperineal biopsy performed using either a grid‐stepper or the original freehand approach without PrecisionPoint.^31^, ^32^, ^33^, ^34^ However, it is worth noting that there was a fairly wide range for each of these values, with overall cancer detection having a range of 43.4–84%, and clinically significant cancer detection having a range of 14–69.1%. Although user technique, experience level, and method used for MRI‐guidance may play a role in this variability, we suspect this likely has more to do with heterogeneity concerning patient characteristics such as pre‐biopsy PSA level, indication for biopsy, and frequency of use of pre‐biopsy MRI. For instance, Szabo et al. reported the lowest CDR, and this is likely attributed to the fact that 44% of the cohort were patients with a prior negative biopsy.^16^ In contrast, Gorin et al.^19^ and Neale et al.^21^ reported the highest CDRs (83.2% and 84%, respectively) and were among the few studies in which all patients had at least one target on MRI. Nevertheless, our subanalysis of biopsy‐naïve patients yielded a CDR for clinically significant disease of 54.5%, which remains favourable compared to other biopsy techniques.

The heterogeneity mentioned above underscores the need for standardized metrics that are less dependent on patient‐level factors to judge biopsy success. One such metric may be the amount of cancer detected normalized by prostate volume (e.g., fraction of positive cores or percent core involvement), with restriction of this analysis to positive cases. For example, in a 2022 study by Urkmez and coworkers, the authors compared the freehand technique with PrecisionPoint to a grid‐based method of transperineal prostate biopsy and included mean number of cores with grade group ≥2 prostate cancer as a readout of diagnostic success.^17^ In this study, the authors found that freehand transperineal prostate biopsy yielded a significantly higher mean number of cores containing clinically‐significant prostate cancer despite fewer obtained biopsy samples.

A well‐known advantage of the transperineal approach to prostate biopsy is the superior ability to sample the anterior prostate.^3^, ^4^, ^5^ Limited sampling of the prostate using the transrectal method is related to two main factors. The first is that the biopsy needle enters the gland from its posterior aspect and must traverse the entire gland before reaching anteriorly. There is a tendency to lose visualization of the needle during this distance of travel, so sampling is biased towards the posterior aspect of the gland. The second reason for undersampling the anterior prostate with the transrectal approach is that sampling of this segment of the gland is only performed with the most distal portion of the biopsy needle (i.e., to avoid puncturing the bladder or the blood vessels on the dorsal aspect of the gland). In contrast, with the transperineal approach, the biopsy needle enters the prostate at its apex and one can easily adjust the height at which the prostate is sampled. The entire length of the throw of the biopsy needle will obtain tissue from this chosen position. The issue of limited anterior sampling of the prostate with the transrectal approach was highlighted in a study by Schouten et al.^35^ In this report, the authors found that approximately 40% of prostate tumours detected on MRI‐targeted biopsy arose in the anterior aspect of the prostate. Importantly, standard systematic transrectal sampling failed to detect these tumours in nearly 80% of cases. In contrast, systematic sampling only missed 20% of posteriorly located tumours.

The efficacy of PrecisionPoint for detecting anteriorly located tumours was apparent in our analysis. In one study, Chen et al. reported an anterior tumour CDR of 49.5% in biopsy‐naïve patients, of which 83.8% were clinically significant.^9^ Additionally, Szabo et al. found that 80% of patients with a positive biopsy after a prior negative biopsy procedure harboured cancer in the anterior region only.^16^ Similarly, in a case–control study of men on surveillance for low‐risk prostate cancer, Meyer et al. found not only that the overall rate of cancer upgrading was higher with the transperineal versus transrectal approach (21.2% vs. 14.7%, *p* = 0.01), but also that the proportion of upgraded tumours was more than twice as likely to arise from the anterior aspect of the prostate (44% vs. 18.7%, *p* = 0.01).^5^

Multiple experiences with the PrecisionPoint device have assessed the relative importance of systematic biopsies. In two studies, 12–14% of clinically significant cancers that were missed on targeted biopsy were detected on systematic sampling of the prostate.^20^, ^21^ Further support for performing systematic biopsies comes from a study by Kim et al., in which up to 18.2% of cancers detected on targeted biopsy were upgraded on systematic biopsy.^13^ Although some reports found that targeted biopsies alone were sufficient to detect clinically significant disease using the transperineal approach, this may be due to sampling of fewer systematic cores contributing to decreased sensitivity.^36^, ^37^ In addition, the added value of systematic biopsy to targeted biopsy may vary depending on the PI‐RADS score of targeted lesions.^21^

In our analysis, the rates of post‐procedural complication for transperineal biopsy with the PrecisionPoint device were similar to those reported for other transperineal techniques.^38^, ^39^ Most notably, the pooled rate for post‐biopsy sepsis was only 0.1%, which is far lower than with the transrectal approach.^1^, ^2^ With the estimated cost of post‐biopsy sepsis ranging from $8672 to $19 100 USD,^40^ increased adoption of the transperineal approach represents a clear opportunity to reduce healthcare expenditures. It is also worth noting that the transperineal approach with the PrecisionPoint device appears to allow for the safe omission of antibiotic prophylaxis, which can further reduce healthcare costs and potentially aid in preventing the emergence of multidrug‐resistant bacteria. More specifically, of the 3129 cases in our analysis where antibiotic use was specified, antibiotic prophylaxis was entirely omitted in 1257 (40%) cases, with no observed increase in the rate of infectious complications. This observation is consistent with other reports regardless of method for performing transperineal prostate biopsy.^41^

One critique of the analysed literature on the PrecisionPoint device is the relative lack of direct comparisons between use of this device and other various methods for performing transperineal prostate biopsy. With that said, the literature has consistently shown a benefit in terms of shorter procedure times with the PrecisionPoint device as compared to grid‐based techniques.^10^, ^17^ Another critique of the present analysis is that the advantages associated with use of PrecisionPoint are likely more attributable to the transperineal approach in general, and not use of the device itself. With that said, within the broad category of freehand techniques for performing transperineal prostate, there is evidence supporting the need for a “device‐driven” approach that incorporates a needle guide that is directly coupled to the ultrasound probe. For example, Pramod et al. found that use of a 3D‐printed, probe‐mounted needle guide resulted in shorter procedural times and improved biopsy accuracy among trainees performing transperineal biopsies in a simulation setting.^42^ Moreover, all participants found the device was easy to use and made the procedure easier to perform. These same benefits should hold true for other probe‐mounted needle guides such as the PrecisionPoint device or similar products from BK Medical ApS (Herlev, Denmark), Hitachi (Tokyo, Japan), and KOELIS, Inc. (Grenoble, France).

The key distinction between the PrecisionPoint device and other probe‐mounted needle guides is the integration of a purpose‐built common access cannula that limits the number of needle sticks to the perineum. Additionally, the device's uniquely engineered access trocar enables dynamic needle re‐directioning throughout the procedure without loss of needle visualization or risk of injury to the tissues. This is possible because of the thick‐walled design of the cannula, which prevents bowing or bending of the needle with passage, as well as the incorporation of a dull bevelled edge at the trocar's distal end. Although use of this disposable device is more costly than other methods, a 2022 market analysis conducted by the National Institute for Health and Care Excellence in the United Kingdom identified PrecisionPoint as one of four transperineal biopsy devices to be a cost‐effective use of healthcare resources.^43^

This review is not without limitations. Importantly, most of the studies in our analysis were retrospective in design. Additionally, the single‐arm nature of most studies limits our ability to make direct comparisons between biopsy with PrecisionPoint and other biopsy methods. Another limitation is that our analysis did not stratify by variation in patient‐level factors such as the use of MRI and indication for biopsy. Although these factors likely explain the wide range of reported CDRs, we believe our pooled averages for overall (68.0%) and clinically significant (42.6%) disease to be very reasonable.

## 5 CONCLUSIONS

The PrecisionPoint device is a safe and effective tool that facilitates the performance of freehand transperineal prostate biopsy. The well‐known advantages of the transperineal technique, such as favourable CDRs and low infectious complication rates, are preserved across published series evaluating this device. In addition, the available literature uniformly shows that transperineal prostate biopsy can be performed under local anaesthesia with satisfactory patient comfort using this novel device. This will likely lead to increased adoption of the transperineal approach by urologists who in the past were deterred from performing this procedure due to the historical need for general anaesthesia with other biopsy methods.

## CONFLICT OF INTEREST

MJA is the inventor of the PrecisionPoint device and co‐owner of Perineologic. MAG is a paid consultant to BK Medical ApS, Perineologic, and KOELIS, Inc. The remaining authors declare no conflicts of interest.

## AUTHOR CONTRIBUTIONS

**Michael A. Gorin:** Conceptualization, Methodology, Validation, Investigation, Resources, Data curation, Writing ‐ Review & editing, Visualization, Supervision, Project administration, Funding acquisition. **Spyridon P. Basourakos:** Methodology, Investigation, Data curation, Writing ‐ Review & editing. **Michael Tzeng:** Methodology, Software, Formal analysis, Investigation, Data curation, Writing ‐ Original draft. **Hiten D. Patel:** Software, Formal analysis, Resources, Writing ‐ Review & editing, Supervision. **Matthew J. Allaway:** Writing ‐ Review & editing. **Jim C. Hu:** Writing ‐ Review & editing.

## Supporting information


**Figure S1.** Preferred reporting items for systematic reviews and meta‐analyses flow diagram. Search string: ((transperineal prostate biopsy) OR (transperineal biopsy)) AND ((freehand) OR (precisionpoint) OR (local anaesthesia)).

**Figure S2.** Images of the PrecisionPoint device being used in conjunction with the UroNav MRI/ultrasound fusion platform (Philips North America Corp., Cambridge, MA). (A) Side and (B) top views of the urologist holding the ultrasound probe equipped with the needle guide. (C) Sagittal view of the biopsy needle being guided to a lesion of interest using with the fusion system.

**Figure S3.** Forest plots for cancer detection rates of (A) overall and (B) clinically significant disease among biopsy‐naïve patients.

**Figure S4.** Forest plots for cancer detection rates of (A) overall and (B) clinically significant disease according to PI‐RADS score for MRI‐targeted lesion. Neale et al. (2020) graded by Likert scale.

**Figure S5.** Assessment of publication bias with funnel plots for (A) overall and (B) clinically significant cancer detection rates.
