# Supplementary material for: Pooled outcomes of performing freehand transperineal prostate biopsy with the PrecisionPoint Transperineal Access System
Source: BJUI Compass. 2022 Jun 28;3(6):434–42. doi: 10.1002/bco2.178 (PMC9579885; doi:10.1002/bco2.178)
Supplement: Supplementary file 1 — Figure S1. Preferred reporting items for systematic reviews and meta‐analyses flow diagram. Search string: ((transperineal prostate biopsy) OR (transperineal biopsy)) AND ((freehand) OR (precisionpoint) OR (local anaesthesia)). [file BCO2-3-434-s004.pptx]

## Slide 1
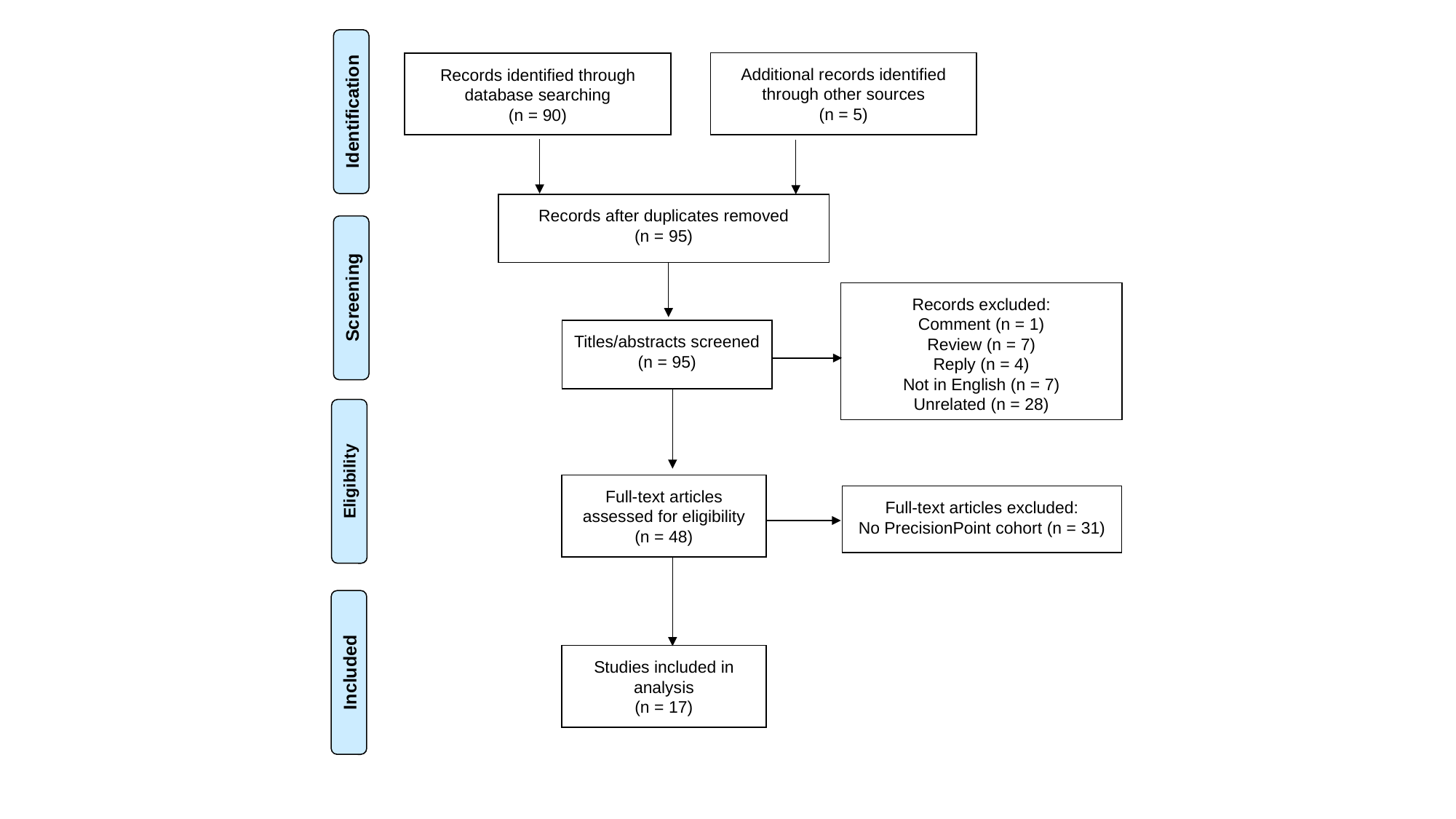

Additional records identified through other sources
(n = 5)
Records identified through database searching
(n = 90)
Identification
Records after duplicates removed
(n = 95)
Screening
Records excluded:
Comment (n = 1)
Review (n = 7)
Reply (n = 4)
Not in English (n = 7)
Unrelated (n = 28)
Titles/abstracts screened
(n = 95)
Eligibility
Full-text articles assessed for eligibility
(n = 48)
Full-text articles excluded:
No PrecisionPoint cohort (n = 31)
Studies included in analysis
(n = 17)
Included
