# Supplementary figures and images for: Pooled outcomes of performing freehand transperineal prostate biopsy with the PrecisionPoint Transperineal Access System
Source: BJUI Compass. 2022 Jun 28;3(6):434–42. doi: 10.1002/bco2.178 (PMC9579885; doi:10.1002/bco2.178)

## Slide 1
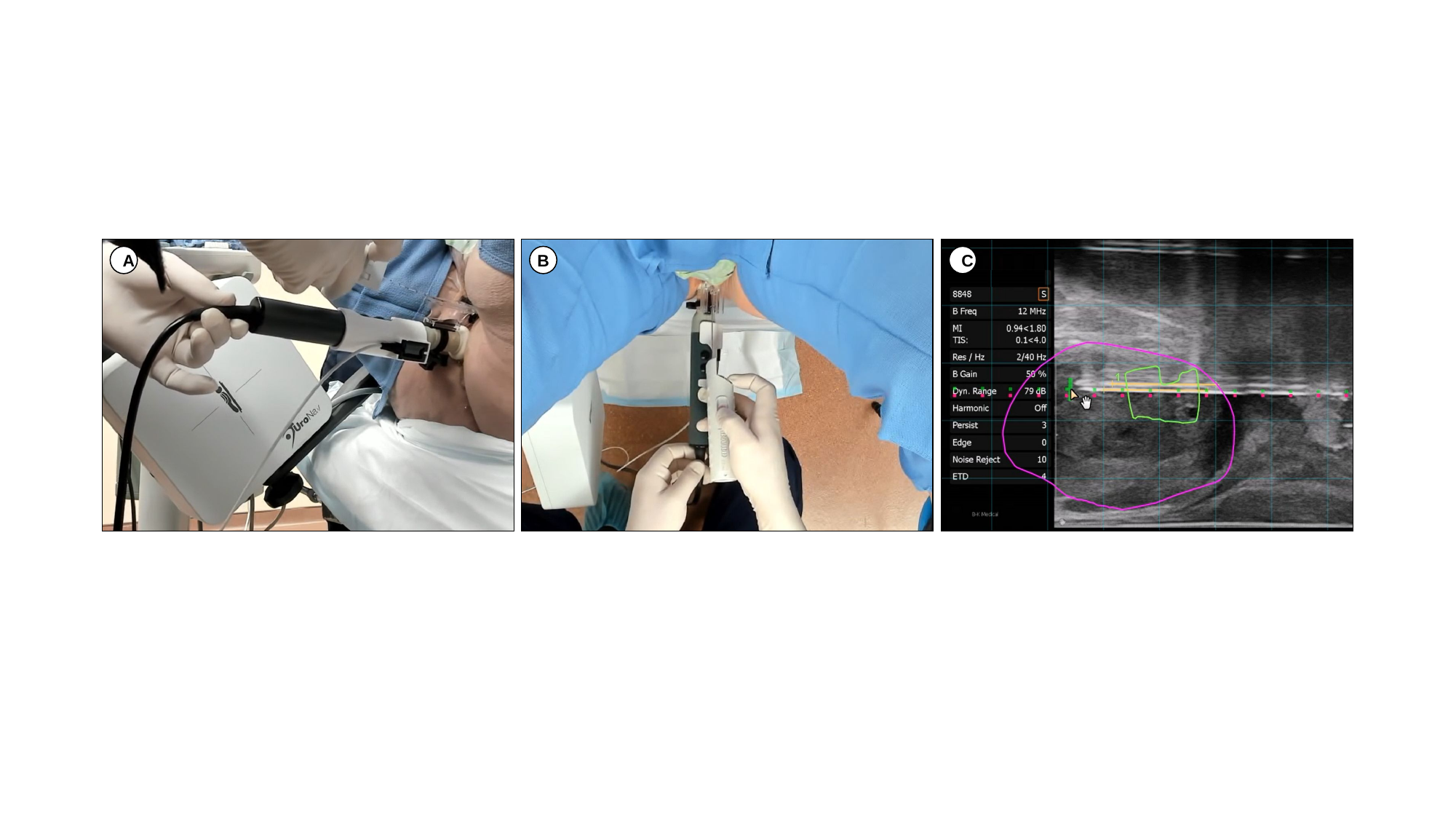

A
B
C

Supplement: Supplementary file 2 — Figure S2. Images of the PrecisionPoint device being used in conjunction with the UroNav MRI/ultrasound fusion platform (Philips North America Corp., Cambridge, MA). (A) Side and (B) top views of the urologist holding the ultrasound probe equipped with the needle guide. (C) Sagittal view of the biopsy needle being guided to a lesion of interest using with the fusion system. [file BCO2-3-434-s001.pptx]

## Slide 1
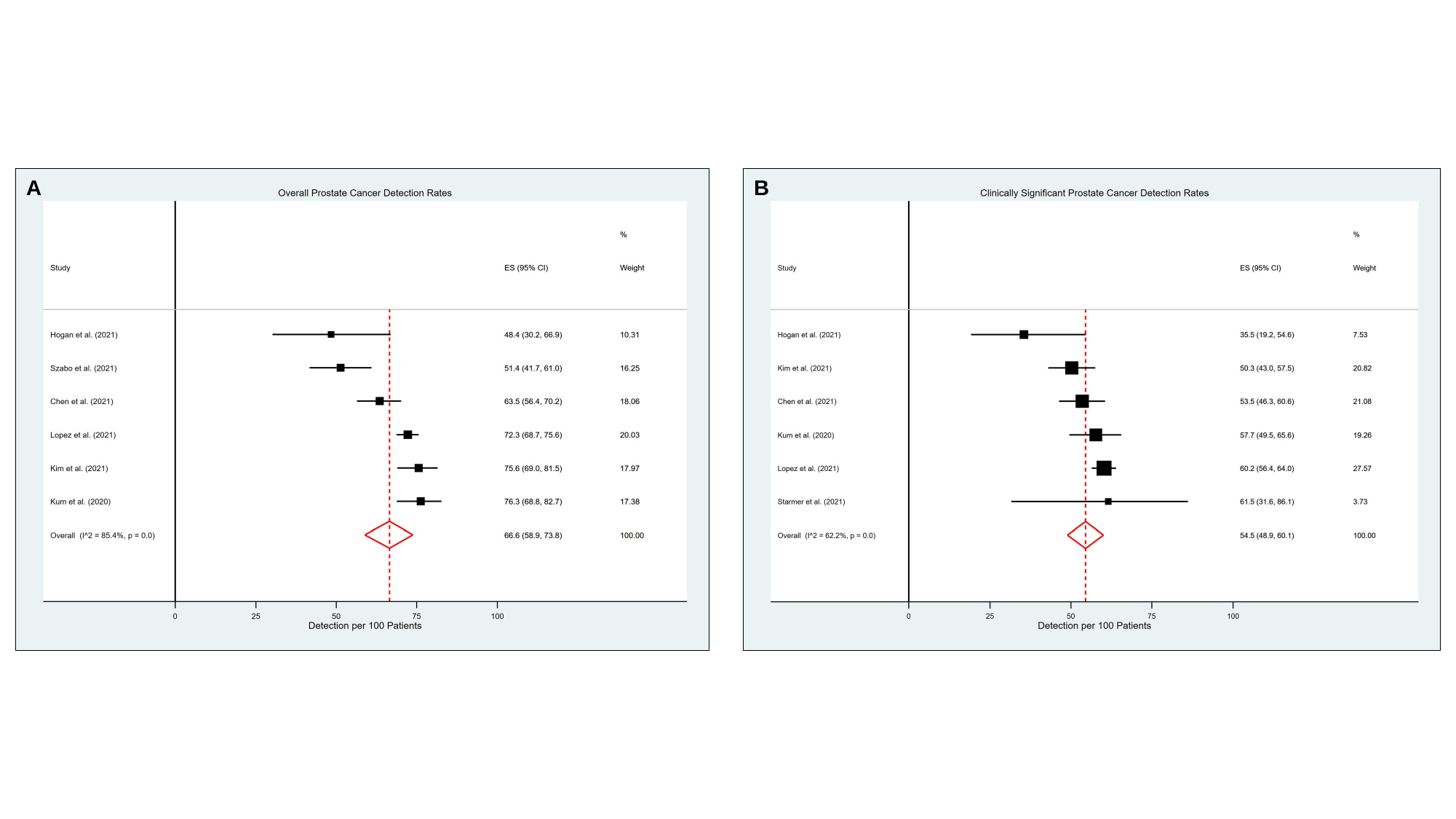

B
A

Supplement: Supplementary file 3 — Figure S3. Forest plots for cancer detection rates of (A) overall and (B) clinically significant disease among biopsy‐naïve patients. [file BCO2-3-434-s003.pptx]

## Slide 1
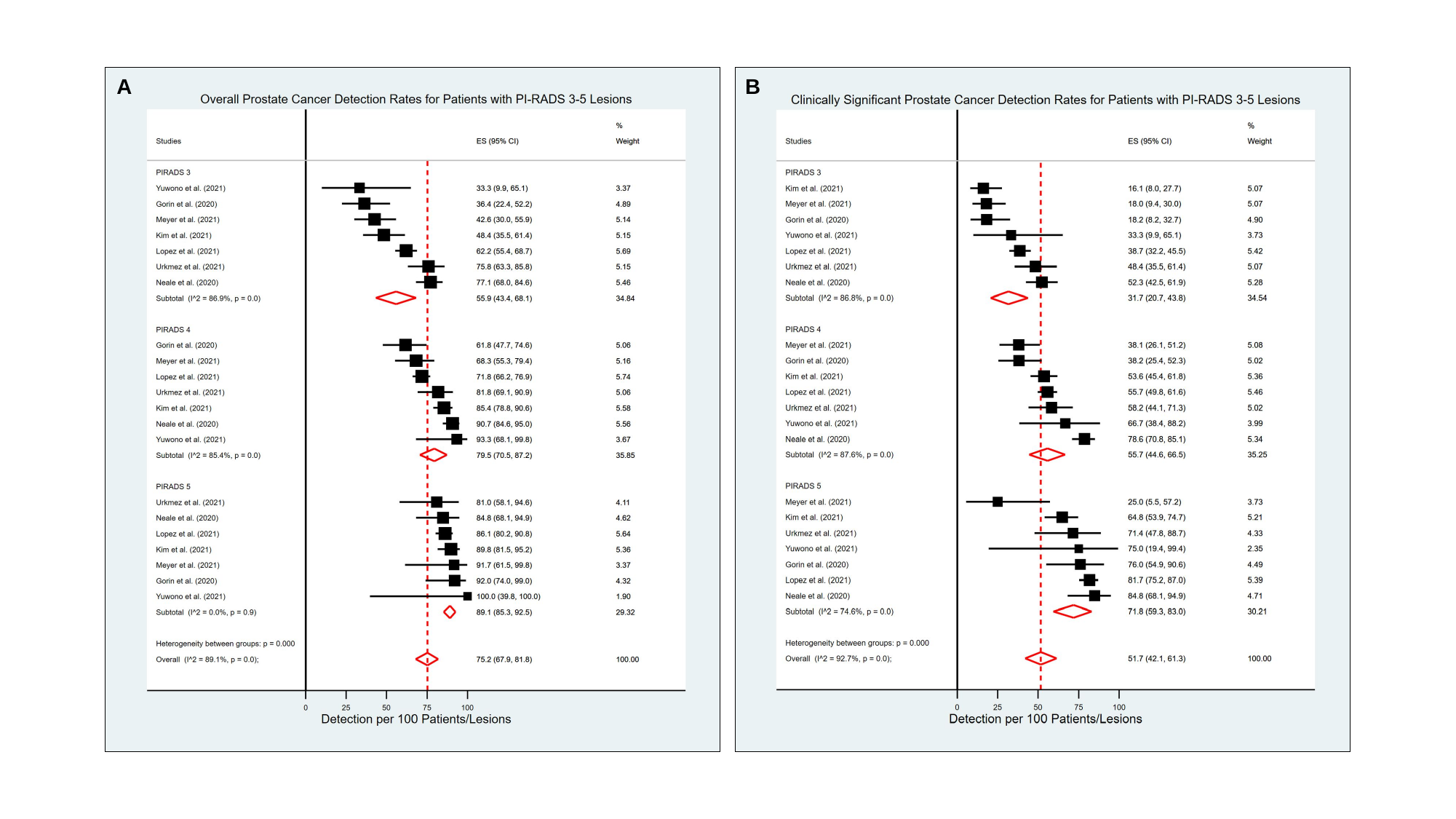

A
B

Supplement: Supplementary file 4 — Figure S4. Forest plots for cancer detection rates of (A) overall and (B) clinically significant disease according to PI‐RADS score for MRI‐targeted lesion. Neale et al. (2020) graded by Likert scale. [file BCO2-3-434-s005.pptx]

## Slide 1
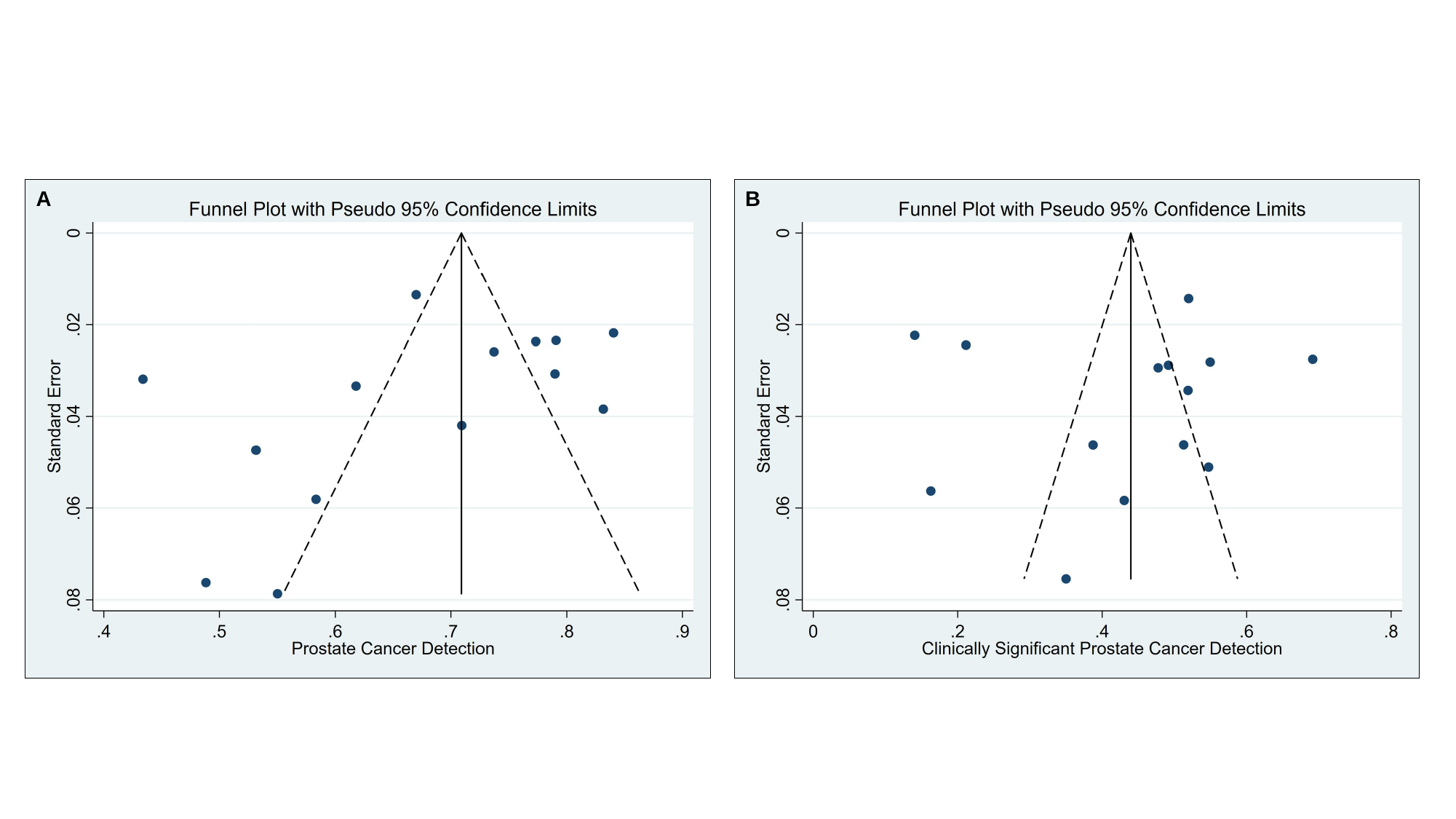

A
B

Supplement: Supplementary file 5 — Figure S5. Assessment of publication bias with funnel plots for (A) overall and (B) clinically significant cancer detection rates. [file BCO2-3-434-s002.pptx]
